# When Is Exposure to a Natural Disaster Traumatic? Comparison of a Trauma Questionnaire and Disaster Exposure Inventory

**DOI:** 10.1371/journal.pone.0123632

**Published:** 2015-04-08

**Authors:** Emily W. Harville, Marni Jacobs, Renée Boynton-Jarrett

**Affiliations:** 1 Department of Epidemiology, Tulane School of Public Health and Tropical Medicine, New Orleans, LA, United States of America; 2 Division of General Pediatrics, Boston University School of Medicine, Boston, Massachusetts, United States of America; Technion - Israel Institute of Technology, ISRAEL

## Abstract

Few studies have compared the sensitivity of trauma questionnaires to disaster inventories for assessing the prevalence of exposure to natural disaster or associated risk for post-disaster psychopathology. The objective of this analysis was to compare reporting of disaster exposure on a trauma questionnaire (Brief Trauma Questionnaire [BTQ]) to an inventory of disaster experience. Between 2011 and 2014, a sample of 841 reproductive-aged southern Louisiana women were interviewed using the BTQ and completed a detailed inventory about exposure to hurricanes and flooding. Post-traumatic stress disorder (PTSD) symptomology was measured with the Post-Traumatic Stress Checklist, and depression with the Edinburgh Depression Scale. The single question addressing disaster exposure on the BTQ had a sensitivity of between 65% and 70% relative to the more detailed questions. Reporting disaster exposure on the BTQ was more likely for those who reported illness/injury due to a hurricane or flood (74%-77%) or danger (77-79%), compared to those who reported damage (69-71%) or evacuation (64-68%). Reporting disaster exposure on the BTQ was associated with depression (odds ratio [OR] 2.29, 95% confidence interval [CI] 1.43-3.68). A single question is unlikely to be useful for assessing the degree of exposure to disaster across a broad population, and varies in utility depending on the mental health outcome of interest: the single trauma question is useful for assessing depression risk.

## Introduction

Assessment of traumatic event exposure is a complex measurement issue, involving issues of definition, assessment methodology, and reporting consistency and validity [1]. A traumatic experience was originally defined as an overwhelming experience outside of the usual range. The DSM-IV criteria for post-traumatic stress disorder (PTSD) redefined traumatic experiences in subjective terms, first, by defining the range of qualifying stressors (A1) and second, by requiring that the person’s response involved intense fear, helplessness, or horror (A2) [2]. The DSM-5 requires identification of certain triggers, whether experienced, witnessed, or happening to a close family member or friend; exposure through media is generally excluded, and the second criterion of subjective response (A2) has been removed [3]. Natural disasters are usually considered traumatic, but in fact result in a range of physical and mental health outcomes [4]. The degree of exposure to a disaster is an important risk factor for developing post-disaster PTSD [5]. More severe and longer lasting mental health outcomes are often associated with events that involve physical injury, witnessing death or injury of others, threat to life, and property loss [4, 6]. Disasters also frequently involve populations who were indirectly or tangentially exposed to the event (for instance, seeing television reports or living in a neighboring, unaffected town) and since this is a larger population, the overall population burden of mental health consequences may be higher in this group, even though the individual risk is lower [7]. Whether such mental health consequences should be considered “true” PTSD is a matter of debate [3].

Estimates of the prevalence of exposure to traumatic events will vary according to the inclusiveness of the stressor criterion and the methods used to measure it [8]. Researchers have found that even with consistent questions and short time periods between measure administrations, report of traumatic events is not completely reliable [1]. Use of a list of events, and the number of events included in the list, have important implications for estimating prevalence. For example, using a list instead of a single question, or a long list as opposed to a short list, yields higher prevalence estimates of trauma exposure and higher estimates of the average number of traumas per exposed person [8]. Because of this, a list of events has become the standard measurement procedure, and has been incorporated into current versions of the major structured interviews [8]. So-called “catch-all” items, such as whether any traumatic event has been experienced, seem to detect events less reliably than items or instruments describing a particular event, where the cues from the list provide a more consistent structure for remembering exposure [1].

Outside of these general principles, head-to-head comparisons of different measurement instruments remain rare. The implications of using a catch-all question (e.g.,“Have you ever experienced a natural or manmade disaster?”) versus using a structured list (e.g., “Was your home damaged in a natural disaster? Did anyone near to you die?”) are not altogether clear, and we are not aware of previous research directly addressing this question for disaster reporting. Some studies have compared different criteria for defining a traumatic experience; for instance, the broader definition of traumatic experiences (incorporating subjective stressors, regardless of whether they were “outside the normal range”) in the DSM-IV led to a larger reported prevalence of traumatic exposures in a community sample, though it did not substantially improve prediction of PTSD relative to the single trauma criterion (A1) [2]. A study of survivors of a shooting in Norway and combat veterans have found no major differences in PTSD prevalence between the DSM-IV and DSM-5 criteria, although the triggers were almost all traumatic under both definitions (100% for the shooting and 87% for combat veterans) [9, 10].

In disaster research, the purpose of the data collection varies depending on the study. Often, a central aim is to assess whether an event was traumatic to the degree likely to produce mental or physical harm. Other types of studies may be more interested in assessing the proportion of the population exposed to a disaster, or a particular aspect of a disaster, such as the proportion that experienced significant property or economic damage. Keeping the interview or questionnaire short is likely to be a goal regardless. In this analysis, we compare report of disaster exposure in a traumatic event inventory (the Brief Trauma Questionnaire [BTQ] [11]) and reports of individual disasters in a highly disaster-exposed population. Specifically, we were interested in two research questions: 1) Did women who reported exposure to a hurricane or flood on a disaster-specific questionnaire also endorse that exposure on the BTQ? and 2) Which instrument (the BTQ or disaster-specific questionnaire) more strongly predicted adverse mental health among those endorsing hurricane/flood exposure? We concentrated on two mental health outcomes: PTSD, as it is the outcome most directly tied to traumatic experience, and depression, one of the most common post-disaster mental health sequelae [12]. We also explored whether the concordance of survey responses varied by participant characteristics, including race, age, and pregnancy status.

## Materials and Methods

### Participants

Pregnant and non-pregnant women of reproductive age were recruited through prenatal, WIC, and general health clinics as well as community organizations in southern Louisiana, for a study of the Deepwater Horizon oil spill, lifetime adversity, and reproductive health. To participate in the study, women needed to be 18–45 years (or older and pregnant) and have lived in the Gulf of Mexico region during 2010, the time of the oil spill. The women in the study were young, majority Black (67%), and low-income (Table 1). Most lived in urban or suburban areas, and the majority did not live directly adjacent to the coast. A large majority (90%) had given birth to at least one child.

**Table 1:** Description of study population (n = 841).

	N*	%
Age		
18–25	253	32.0
>25–30	227	28.7
>30–35	156	19.8
>35	154	19.5
Race		
white	210	26.1
black	535	66.5
other	59	7.3
Education		
High school or less	406	50.8
some college/Associate's degree	336	42.0
college graduate or higher	58	7.3
Income		
<$15,000	364	45.8
$15–25,000	264	33.2
> = $25,000	167	21.0
Pregnant at time of interview		
yes	232	27.6
no	609	72.4
Marital status		
married	187	22.9
living with partner	105	12.9
single, divorced, widowed	524	64.2
Parity		
parous	532	90.0
nulliparous	59	10.0
Area of residence		
urban/suburban	705	91.2
rural	68	8.8
Proximity to coast		
ZIP code proximal to coast	178	22.9
further from coast	601	77.2

*data may not add to 841 due to missing data

### Measures

Trauma history was measured using questions from a modified version of the Brief Trauma Questionnaire (BTQ;[11, 13, 14]). The BTQ was derived from the Brief Trauma Interview (Schnurr et al., 1999), a clinician-administered 10-item interview based on the Trauma Assessment for Adults [15]. The measure was developed to be a sensitive screening tool for determining whether an individual has experienced a traumatic event that meets both the A1 and A2 criterion required for a diagnosis of PTSD according to the DSM-IV. The measure evaluates a number of potentially traumatic life events including motor vehicle accidents, death of a close friend or family member, and life-threatening illnesses. Natural disasters are also assessed through the question: “Have you ever experienced a natural or human made disaster (such as fire, hurricane, flood, terrorist attack)?” Kappa coefficients for all event-specific items range from 0.74 to 1.00 [16]. While psychometric data for the BTQ is currently limited, interrater reliability has been shown to be good to excellent for all of the primary trauma categories, and criterion validity has been demonstrated repeatedly, with expected associations found between BTQ measured trauma and PTSD symptom severity [17, 18].

#### Disaster exposure

Hurricane/flood experience was measured with 12 questions (S1 Table), based on a study of Hurricane Andrew by Kaniasty and Norris [19]. Women were asked individually about Hurricanes Katrina, Rita, Ike, Gustav, and the Mississippi flooding of 2010. Hurricane Isaac, which hit in 2012, was added during data collection. This measure has been associated with poorer mental health and birth outcomes in previous studies [20, 21] and factor analysis has been conducted [22] to group the questions for similar aspects of exposure, creating three disaster exposure categories: damage (some or more “damage to house”, “house flooded”, some or greater “impact of hurricane”, and some or greater “total impact on belongings of other people”), perceived/experienced danger (“felt life in danger”, “walked in floodwater”, and “saw someone die”), and illness/injury to self or others (“experienced illness/injury”, “someone in household experienced illness/injury”, “someone near died”, and “someone else important experienced illness/injury”). An additional question asked whether the respondent had evacuated for the disaster.

Natural disaster exposure was examined for each disaster individually as well as across disasters, in the following ways: one, any exposure to a given event; two, any exposure to a disaster exposure category (endorsing any one of the sub-items); three, number of exposures to a category (danger, damage, illness) across disasters; and four, number of total events experienced. For the third categorization, someone who had a severe exposure during one disaster only could have a high score, while for the fourth categorization, only someone who had been exposed during multiple events would have a high score.

#### Mental health

The PCL is the Post-Traumatic Checklist, which asks about symptoms related to any stressful experience and is based on the DSM-IV criteria for PTSD [23]. PTSD symptoms were dichotomized at scores greater than 50, which has been shown to perform well as a cut-off relative to clinical diagnosis [23, 24]. The Edinburgh Postnatal Depression Index (EDS) was used to assess symptoms of depression. This scale was originally designed to address postnatal depression, but has been validated for use in pregnant and non-pregnant samples [25–28]. Probable depression was defined as EDS score greater than 12, which is estimated as the best cut-off for indicating likely depression [29].

### Procedure

Trained research assistants interviewed the participants about their traumatic events across their life and their mental health. Women also completed a questionnaire which included information on disaster exposure; this questionnaire was normally completed just after the interview. 1046 women were recruited by the time of this analysis; 841 had information on both the self-reported disaster exposure and the BTQ. The majority of the loss to follow-up was due to women who did not complete the questionnaire. Women included in this analysis did not differ from the overall sample with respect to race, education, income, or area of residence, but they were less likely to be partnered (married or living with a partner) or pregnant, and were on average one year older (mean difference 1.2 years, p = 0.04).

### Data analysis

The number of women indicating exposure to disaster on the BTQ, exposure to individual disasters, and exposure to individual aspects of disaster was calculated. The sensitivity of the BTQ question relative to the disaster exposure measure was calculated, with a 95% confidence interval (CI) as the CI for a binomial proportion. Variation in sensitivity by race, age, marital status, income, education, pregnancy status, and location was assessed using chi-square tests.

Logistic regression was used to assess the ability of the scales to predict PTSD and depression. The area under the receiver operating curve (aROC) was calculated to assess predictive value, and the area under the curves contrasted for the different measures, using the proc logistic/roc and roccontrast statements in SAS version 9.3. We then examined the predictive value adjusted for covariates associated with predictor and outcome (age, race, income, and pregnancy status). Multiple imputation was used to account for missing data in the covariates.

The Institutional Review Boards of Ochsner Clinic, Chabert Hospital, the Louisiana Department of Health and Human Services, and Tulane University approved this study, and all women provided written informed consent.

## Results and Discussion

Five hundred and fifty-one (70%) of the women reported disaster exposure on the BTQ, while 791 (94% of those answering those questions) reported at least one indicator of exposure to Hurricane Katrina, 550 (68%) reported exposure to Rita, 580 (72%) reported exposure to Gustav, 397 (72%) reported exposure to Ike, 219 (27%) reported exposure to Mississippi flooding, and 449 (65%) reported exposure to Isaac. This corresponds to sensitivity of between 65% and 70% for the BTQ (Table 2). 246 (35%) of women reported that a hurricane, flood, or natural disaster was the most traumatic experience they had experienced in their lifetime. Sensitivity of the BTQ was close to 100% for these women.

**Table 2:** Sensitivity of the Brief Trauma Questionnaire in identifying any exposure to a hurricane.

	N reporting any disaster exposure on the Brief Trauma Questionnaire	N reporting on disaster inventory	% (Se)	95% CI
Katrina	551	791	70	66–73
Rita	375	550	68	64–72
Gustav	386	580	67	63–70
Ike	267	397	67	62–72
Mississippi floods	143	219	65	59–72
Isaac	305	449	68	63–72

For domains of hurricane experience, reporting on the BTQ was somewhat more likely for those who reported illness/injury/death (74%-77%) or exposure to danger (77–79%), compared to those who reported damage (69–70%) or evacuation (64–68%) (Table 3). Sensitivity of the BTQ was substantially lower for number of experiences of disaster: 60% for women who reported experiencing all 6 natural disasters. Limiting the exposure to those with higher degrees of exposure did not consistently increase the sensitivity of the BTQ.

**Table 3:** Variation in traumatic experience reporting by experience of a natural disasters in Southern Louisiana women, N = 841.

	N reporting any disaster exposure on the Brief Trauma Questionnaire	N reporting on disaster	% (Se)	95% CI
across disasters				
any illness/injury	288	368	78	74–82
any damage	543	781	70	66–73
any danger	453	597	76	72–79
evacuated	484	712	68	64–71
high number of total experiences, regardless of number of disasters				
illness (4+)	81	105	77	68–85
damage (10+)	181	259	70	64–75
danger (5+)	116	147	79	71–85
Number of discrete disaster events experienced				
overall (6)	96	161	60	52–67
illness (3+)	55	74	74	63–84
damage (4+)	131	191	69	61–75
danger (3+)	189	245	77	71–82
evacuation (4+)	161	253	64	57–70

There was a tendency for the BTQ to be more sensitive in the oldest women. This was especially clear for experiencing any damage (sensitivity in the youngest women 0.63, 0.56–0.69; oldest 0.79, 0.71–0.85); or any evacuation (youngest 0.61, 0.54–0.67; oldest: 0.79, 0.71–0.86). When individual disasters were examined, this held especially for Katrina (e.g, for any Katrina exposure, sensitivity for the youngest women was 0.63, 0.56–0.69; oldest 0.78, 0.70–0.85, p<0.05 for difference; p<0.05 also for damage and evacuation due to Katrina). Sensitivity did not vary by other covariates.

Overall prevalence in the sample of PTSD was 6.7% and of depression was 16.0%. Reporting disaster exposure on the BTQ was associated with depression (OR 2.29, 1.43–3.68) though less strongly with PTSD (OR 1.64, 0.84–3.18) (Table 4). This made it a better predictor of depression than dichotomous exposure to any specific disaster (ORs 1.03 [any exposure to Rita, p for difference in aROC = 0.03] to 1.83 [any exposure to Isaac, p for difference in aROC = 0.49]). Several disaster characteristics were strongly associated with mental health outcomes. Disaster-associated illness/injury and danger were consistently more strongly associated with both PTSD and depression than the BTQ (higher ORs), but the aROCs generally did not differ. Odds ratios for associations with damage were high but imprecise, while evacuation for any disaster was unassociated with either PTSD or depression. The aROC for depression was higher for the BTQ than for evacuation, and sometimes for damage, when the individual hurricanes were examined (S3 Table).

**Table 4:** Prediction of mental illness by indicators of disasters relative to the Brief Trauma Questionnaire in southern Louisiana women, adjusted for covariates.

			PTSD		p for difference			depression		p for difference
	OR*	95% CI	aROC	CI		OR*	95% CI	aROC	CI	
BTQ	1.64	0.84–3.18	0.61	0.51–0.70		2.29	1.43–3.68	0.64	0.59–0.69	
Katrina	1.06	0.31–3.58	0.61	0.52–0.69	0.65	1.81	0.69–4.71	0.60	0.55–0.66	0.06
Rita	1.42	0.74–2.72	0.60	0.52–0.69	0.77	1.03	0.68–1.56	0.59	0.64–0.65	0.03
Gustav	1.89	0.93–3.85	0.63	0.55–0.71	0.59	1.63	1.03–2.57	0.61	0.56–0.66	0.20
Ike	2.83	1.52–5.28	0.67	0.59–0.75	0.11	1.53	1.04–2.26	0.61	0.55–0.66	0.23
Mississippi	1.16	0.63–2.14	0.61	0.52–0.69	0.76	1.52	1.02–2.29	0.61	0.56–0.67	0.22
Isaac	1.86	0.90–3.85	0.63	0.54–0.73	0.50	1.83	1.13–2.95	0.63	0.57–0.68	0.49
overall (across disasters)										
any illness	2.84	1.57–5.14	0.67	0.59–0.75	0.08	2.95	1.97–4.40	0.68	0.63–0.73	0.21
any damage	3.59	0.48–26.63	0.63	0.55–0.71	0.62	2.41	0.85–6.85	0.62	0.56–0.67	0.15
any danger	5.23	1.86–14.73	0.68	0.61–0.75	0.02	2.64	1.58–4.44	0.65	0.60–0.70	0.81
evacuated	0.78	0.34–1.80	0.61	0.53–0.70	0.79	0.89	0.49–1.63	0.60	0.54–0.65	0.04
number of total experiences, regardless of number of disasters										
illness										
0	1					1				
1	1.48	0.60–3.67				1.80	1.01–3.19			
2	2.24	1.02–4.92				2.77	1.65–4.64			
3+	5.66	2.80–11.43	0.65	0.56–0.74	0.26	5.14	3.04–8.66	0.65	0.60–0.70	0.79
damage										
0	1					1				
1	2.72	0.35–21.10				1.97	0.67–5.79			
2	3.08	0.39–24.11				2.09	0.70–6.23			
3+	5.28	0.69–40.29	0.65	0.56–0.73	36	3.43	1.17–10.04	0.63	0.57–0.68	0.52
danger										
0	1					1				
1	3.75	1.24–11.37				2.14	1.21–3.81			
2	5.29	1.71–16.31				2.29	1.24–4.25			
3+	8.44	2.77–25.69	0.63	0.54–0.72	0.64	4.40	2.40–8.08	0.64	0.58–0.69	0.79
Number of discrete disaster events experienced										
overall										
0						1				
1	†					1.74	0.21–14.56			
2	1.19	0.37–3.88				2.23	0.27–18.16			
3	1.05	0.32–3.42				3.39	0.42–27.01			
4	2.69	0.92–7.85				3.14	0.39–25.34			
5	2.19	0.74–6.46				2.79	0.35–22.57			
6	1.78	0.61–5.23	0.61	0.53–0.69	0.75	3.94	0.50–31.31	0.61	0.56–0.66	0.16
illness										
0	1					1				
1	2.73	0.84–8.88				2.06	1.13–3.76			
2	7.00	2.26–21.67				2.54	1.34–4.84			
3+	6.70	2.28–19.71	0.64	0.56–0.73	0.38	3.31	1.88–5.82	0.64	0.59–0.70	0.99
damage										
0	1					1				
1	2.68	0.35–20.38				1.92	0.66–5.54			
2	3.45	0.42–28.12				3.23	1.07–9.80			
3+	6.08	0.79–46.64	0.65	0.57–0.73	0.38	3.16	1.07–9.38	0.64	0.59–0.69	0.81
danger										
0	1					1				
1	1.70	0.82–3.52				1.92	1.19–3.09			
2	3.53	1.51–8.28				4.19	2.31–7.62			
3+	6.34	2.93–13.69	0.64	0.56–0.73	0.41	6.06	3.37–10.91	0.61	0.55–0.66	0.13
evacuation										
0	1					1				
1	0.90	0.35–2.34				0.78	0.38–1.57			
2	0.97	0.36–2.62				0.84	0.40–1.75			
3	0.56	0.19–1.66				0.96	0.47–1.98			
4+	0.73	0.29–1.85	0.61	0.53–0.70	0.81	0.97	0.50–1.87	0.60	0.55–0.66	0.06

BTQ, Brief Trauma Questionnaire; OR, odds ratio; CI, confidence interval; aROC, area under the receiver operating curve

*Adjusted for age, pregnancy status, race, and income.

^†^ relative to 1 experience; too few cases in the 0 category to converge.

Researchers and clinicians will have varying goals in assessing disaster exposure.

### Estimating the prevalence of disaster exposure

Measurement of the prevalence of trauma exposure is a topic of some controversy [30]: some argue that disaster exposure only need be measured if it involves significant property loss, injury, or threat of death, while others suggest that any exposure is relevant to mental health [6]. Studies of the health effects of disaster often use area of residence as a proxy for exposure, with no consideration of the details of individual-level exposure [31, 32]. We found that the BTQ question on disaster exposure was fairly sensitive for picking up severe disaster exposure, but had poor sensitivity for more mild disaster exposure. This suggests that a single question is unlikely to be useful for assessing the degree of exposure to disaster across a broad population, especially for those with less direct exposure [7]. The usefulness of a measurement instrument may also vary by the participant’s age at the disaster; we found that sensitivity of the single item was better for older women, especially for Katrina. Participants could have been as young as 8 or 9 for Katrina and children may have been less aware of financial issues due to the disaster or effects on people they did not know.

### Predicting psychological impacts

When the goal was to assess disaster trauma that is related to mental health, the BTQ performed better than an event-specific inventory. Responding ‘yes’ to the BTQ disaster question was associated with depression (though PTSD was better predicted by more detailed questions, particularly those addressing illness or danger). Higher cumulative disaster-related illnesses or dangers had a graded association with depression and PTSD; Disaster exposure leading to damage or evacuation was not strongly related to mental health and less likely to be picked up by the BTQ. This is consistent with prior disaster literature that suggests a measurement procedure that generates a lower prevalence of exposure, such as a single question, will yield a higher conditional risk for adverse mental health, compared with procedures that yield a higher prevalence of exposure, such as a list of events, which enhance the recall of events that are less distinctive [8], and also supports the DSM-5 revisions to the definition of PTSD which require that the trigger involve actual or threatened injury or death [3]. For studies assessing the effects of disaster on mental health, a single question may be sufficient, though more detail assists in determining which aspects of the experience are particularly distressing. Our results are also consistent with previous work suggesting it is the characteristics of disaster exposure, rather than the type of disaster or exposure to a specific disaster (e.g., Hurricane Katrina rather than Hurricane Gustav), that are related to mental health outcomes [6].

## Conclusions

Our population is female, fairly young, and lives in an area of the world that has been exposed to several disasters in recent years; therefore, these results may not be generalizable to all populations. Generally, women are more prone to post-disaster psychopathology than men [12, 33], and studies suggest that the effect of disaster on mental health declines with age [12]. We cannot provide any information about men, and the study did indicate some differences by age, even within the restricted age range. This population was recruited through convenience sampling at a broad range of health and community facilities, which likely makes it more representative of exposure and effects in the community compared to studies of litigants or those referred for clinical evaluation [4]. Also, the multiple disasters that have hit the region mean that self-report of disaster is not limited to a single event, but a more general understanding of disaster. While Hurricane Katrina had the strongest effect on the New Orleans area, other parts of the study area were more affected by Gustav or Ike. Time since the disaster did not appear to be an important predictor of the performance of the scales, as sensitivity was not higher for the more recent events.

Theoretically, the study design allowed for some people to have moved in to the area after Hurricanes Katrina, Rita, and Ike, but given that 95% of the study population was exposed to Katrina, it does not seem that this was a major source of variation in the sample. Multiple comparisons are an issue, although the patterns of results are very similar across disasters and different ways of categorization. The data were collected cross-sectionally; those who report PTSD or depressive symptoms may also be more likely to report disaster exposure. However, there is no reason to believe that the overreporting would be differential for one instrument relative to the other.

The measurement tool and the goals of the study need to be carefully matched when post-disaster research is being conducted. Consideration needs to be given to whether the most important issue is degree of disaster exposure, a particular type of exposure, or self-perception of the importance of an event. Such considerations apply both to disaster researchers and to clinicians assessing lifetime trauma.

## Supporting Information

S1 Table Exposure to disaster scale (adapted from Kaniasty and Norris). (DOCX)

S2 Table Variation in traumatic experience reporting by experience of a hurricane in Southern Louisiana women, N = 841. (DOCX)

S3 Table Prediction of mental health outcomes with adjustment for confounders. (DOCX)
