# Supplementary material for: When Is Exposure to a Natural Disaster Traumatic? Comparison of a Trauma Questionnaire and Disaster Exposure Inventory
Source: PLoS One. 2015 Apr 8;10(4):e0123632. doi: 10.1371/journal.pone.0123632 (PMC4390192; doi:10.1371/journal.pone.0123632)
Supplement: S1 Table — (DOCX) [file pone.0123632.s001.docx]

Table S1. Exposure to disaster scale (adapted from Kaniasty and Norris)

|  | Katrina | Rita | Gustav | Ike | Mississippi flooding | Isaac (added 2012) |
| --- | --- | --- | --- | --- | --- | --- |
| HE1. Did you ever feel like your life was in danger when the hurricane/flood struck or during the aftermath? | Yes  No | Yes  No | Yes  No | Yes  No | Yes  No | Yes  No |
| HE2. Did the hurricane/flood cause you to have an illness or injury? | Yes  No | Yes  No | Yes  No | Yes  No | Yes  No | Yes  No |
| HE3. Did hurricane/flood cause some other member of your household to have an illness or injury? | Yes  No | Yes  No | Yes  No | Yes  No | Yes  No | Yes  No |
| HE4. Did you walk through floodwaters? | Yes  No | Yes  No | Yes  No | Yes  No | Yes  No | Yes  No |
| HE5. Were any family members not living with you injured by the hurricane? | Yes  No | Yes  No | Yes  No | Yes  No | Yes  No | Yes  No |
| HE6. To what extent did the hurricane/flood damage your home? | Enormous  Much  Some  Just a little  None | Enormous  Much  Some  Just a little  None | Enormous  Much  Some  Just a little  None | Enormous  Much  Some  Just a little  None | Enormous  Much  Some  Just a little  None | Enormous  Much  Some  Just a little  None |
| HE7. Did you get floodwaters in your house? | Yes  No | Yes  No | Yes  No | Yes  No | Yes  No | Yes  No |
| HE8. Did you evacuate? | Yes  No | Yes  No | Yes  No | Yes  No | Yes  No | Yes  No |
| HE9. Did you lose belongings that were/will be expensive to replace? | Yes  No | Yes  No | Yes  No | Yes  No | Yes  No | Yes  No |
| HE10. Did you lose anything of sentimental value such as photographs or keepsakes? | Yes  No | Yes  No | Yes  No | Yes  No | Yes  No | Yes  No |
| HE11. Did the hurricane/flood damage your car, truck, or boat? | Yes  No | Yes  No | Yes  No | Yes  No | Yes  No | Yes  No |
| HE12. Which of the following statements best describes the impact of this hurricane/flood on the property and belongings of other family members living in the area? | Enormous  Much  Some  Just a little  None | Enormous  Much  Some  Just a little  None | Enormous  Much  Some  Just a little  None | Enormous  Much  Some  Just a little  None | Enormous  Much  Some  Just a little  None | Enormous  Much  Some  Just a little  None |
| HE13. Did anyone close to you die in the hurricane/flood? | Yes  No | Yes  No | Yes  No | Yes  No | Yes  No | Yes  No |
| HE14. Did you see anyone die in the hurricane/flood? | Yes  No | Yes  No | Yes  No | Yes  No | Yes  No | Yes  No |
| HE15. Did you have a pet die in the hurricane/flood? | Yes  No | Yes  No | Yes  No | Yes  No | Yes  No | Yes  No |
