# Supplementary material for: When Is Exposure to a Natural Disaster Traumatic? Comparison of a Trauma Questionnaire and Disaster Exposure Inventory
Source: PLoS One. 2015 Apr 8;10(4):e0123632. doi: 10.1371/journal.pone.0123632 (PMC4390192; doi:10.1371/journal.pone.0123632)
Supplement: S2 Table — (DOCX) [file pone.0123632.s002.docx]

| Table S2. Variation in traumatic experience reporting by experience of a hurricane in Southern Louisiana women, N=841 | | | | | | |
| --- | --- | --- | --- | --- | --- | --- |
|  |  |  |  |  |  |  |
|  |  | N reporting any disaster exposure on the Brief Trauma Questionnaire |  | N reporting on disaster | % (Se) | 95% CI |
| Katrina |  |  |  |  |  |  |
| any illness |  | 263 |  | 336 | 78 | 74-83 |
| any damage |  | 517 |  | 742 | 70 | 66-73 |
| any danger |  | 409 |  | 533 | 77 | 73-80 |
| evacuated |  | 420 |  | 630 | 67 | 63-70 |
|  |  |  |  |  |  |  |
| Rita |  |  |  |  |  |  |
| any illness |  | 84 |  | 111 | 76 | 67-83 |
| any damage |  | 270 |  | 393 | 69 | 64-73 |
| any danger |  | 170 |  | 223 | 76 | 70-82 |
| evacuated |  | 276 |  | 418 | 66 | 61-71 |
|  |  |  |  |  |  |  |
| Gustav |  |  |  |  |  |  |
| any illness |  | 64 |  | 81 | 79 | 69-87 |
| any damage |  | 217 |  | 308 | 70 | 65-75 |
| any danger |  | 182 |  | 240 | 76 | 70-81 |
| evacuated |  | 294 |  | 452 | 65 | 60-69 |
|  |  |  |  |  |  |  |
| Ike |  |  |  |  |  |  |
| any illness |  | 33 |  | 45 | 73 | 58-85 |
| any damage |  | 134 |  | 193 | 69 | 62-76 |
| any danger |  | 126 |  | 163 | 77 | 70-83 |
| evacuated |  | 165 |  | 262 | 63 | 57-69 |
|  |  |  |  |  |  |  |
| Mississippi |  |  |  |  |  |  |
| any illness |  | 25 |  | 36 | 69 | 52-84 |
| any damage |  | 58 |  | 89 | 65 | 54-75 |
| any danger |  | 71 |  | 96 | 74 | 64-82 |
| evacuated |  | 65 |  | 112 | 58 | 48-67 |
|  |  |  |  |  |  |  |
| Isaac |  |  |  |  |  |  |
| any illness |  | 54 |  | 78 | 69 | 58-79 |
| any damage |  | 199 |  | 301 | 66 | 60-71 |
| any danger |  | 182 |  | 249 | 73 | 67-79 |
| evacuated |  | 146 |  | 240 | 61 | 54-67 |
