# Supplementary material for: When Is Exposure to a Natural Disaster Traumatic? Comparison of a Trauma Questionnaire and Disaster Exposure Inventory
Source: PLoS One. 2015 Apr 8;10(4):e0123632. doi: 10.1371/journal.pone.0123632 (PMC4390192; doi:10.1371/journal.pone.0123632)
Supplement: S3 Table — (DOCX) [file pone.0123632.s003.docx]

| Table S3. Prediction of mental health outcomes with adjustment for confounders. | | | | | | | | | | | | | | | | | |  |  |  |  |  |  |  |
| --- | --- | --- | --- | --- | --- | --- | --- | --- | --- | --- | --- | --- | --- | --- | --- | --- | --- | --- | --- | --- | --- | --- | --- | --- |
|  |  | PTSD | | | | | | |  | depression | | | | | | |  | |  |  |  |  |  |  |
|  |  | OR* | 95% CI |  | aROC | 95% CI |  | p for contrast relative to BTQ |  | OR* | 95% CI |  | aROC | 95% CI |  | p for contrast relative to BTQ |  | |  |  |  |  |  |  |
| Katrina |  |  |  |  |  |  |  |  |  |  |  |  |  |  |  |  |  | |  |  |  |  |  |  |
| any illness |  | 2.82 | 1.58-5.06 |  | 0.67 | 0.59-0.75 |  | 0.11 |  | 2.83 | 1.90-4.21 |  | 0.67 | 0.62-0.72 |  | 0.33 |  | |  |  |  |  |  |  |
| any damage |  | 1.44 | 0.50-4.12 |  | 0.62 | 0.53-0.70 |  | 0.68 |  | 1.38 | 0.70-2.70 |  | 0.60 | 0.55-0.66 |  | 0.05 |  | |  |  |  |  |  |  |
| any danger |  | 2.55 | 1.26-5.19 |  | 0.66 | 0.58-0.73 |  | 0.15 |  | 1.85 | 1.20-2.86 |  | 0.63 | 0.58-0.68 |  | 0.62 |  | |  |  |  |  |  |  |
| evacuated |  | 0.50 | 0.27-0.94 |  | 0.64 | 0.56-0.72 |  | 0.80 |  | 0.97 | 0.60-1.57 |  | 0.60 | 0.54-0.65 |  | 0.04 |  | |  |  |  |  |  |  |
|  |  |  |  |  |  |  |  |  |  |  |  |  |  |  |  |  |  | |  |  |  |  |  |  |
| Rita |  |  |  |  |  |  |  |  |  |  |  |  |  |  |  |  |  | |  |  |  |  |  |  |
| any illness |  | 3.49 | 1.87-6.51 |  | 0.64 | 0.56-0.73 |  | 0.389 |  | 3.12 | 1.96-4.98 |  | 0.64 | 0.59-0.70 |  | 0.87 |  | |  |  |  |  |  |  |
| any damage |  | 1.46 | 0.82-2.61 |  | 0.62 | 0.53-0.70 |  | 0.84 |  | 1.21 | 0.82-1.78 |  | 0.60 | 0.54-0.65 |  | 0.05 |  | |  |  |  |  |  |  |
| any danger |  | 1.72 | 0.96-3.10 |  | 0.62 | 0.53-0.71 |  | 0.84 |  | 1.67 | 1.11-2.50 |  | 0.61 | 0.56-0.67 |  | 0.28 |  | |  |  |  |  |  |  |
| evacuated |  | 0.92 | 0.51-1.66 |  | 0.63 | 0.54-0.71 |  | 0.84 |  | 0.88 | 0.60-1.31 |  | 0.60 | 0.54-0.65 |  | 0.06 |  | |  |  |  |  |  |  |
|  |  |  |  |  |  |  |  |  |  |  |  |  |  |  |  |  |  | |  |  |  |  |  |  |
| Gustav |  |  |  |  |  |  |  |  |  |  |  |  |  |  |  |  |  | |  |  |  |  |  |  |
| any illness |  | 4.58 | 2.34-8.98 |  | 0.65 | 0.56-0.74 |  | 0.24 |  | 3.92 | 2.32-6.62 |  | 0.64 | 0.59-0.70 |  | 0.94 |  | |  |  |  |  |  |  |
| any damage |  | 2.68 | 1.50-4.81 |  | 0.67 | 0.59-0.75 |  | 0.24 |  | 1.95 | 1.33-2.87 |  | 0.63 | 0.57-0.68 |  | 0.74 |  | |  |  |  |  |  |  |
| any danger |  | 2.80 | 1.59-4.93 |  | 0.67 | 0.59-0.75 |  | 0.17 |  | 2.18 | 1.47-3.22 |  | 0.65 | 0.60-0.70 |  | 0.85 |  | |  |  |  |  |  |  |
| evacuated |  | 0.98 | 0.54-1.77 |  | 0.62 | 0.53-0.71 |  | 0.44 |  | 1.15 | 0.77-1.73 |  | 0.60 | 0.55-0.66 |  | 0.06 |  | |  |  |  |  |  |  |
|  |  |  |  |  |  |  |  |  |  |  |  |  |  |  |  |  |  | |  |  |  |  |  |  |
| Ike |  |  |  |  |  |  |  |  |  |  |  |  |  |  |  |  |  | |  |  |  |  |  |  |
| any illness |  | 5.31 | 2.44-11.54 |  | 0.62 | 0.53-0.70 |  | 0.88 |  | 3.76 | 1.97-7.20 |  | 0.62 | 0.56-0.67 |  | 0.34 |  | |  |  |  |  |  |  |
| any damage |  | 2.54 | 1.42-4.53 |  | 0.66 | 0.57-0.74 |  | 0.36 |  | 1.71 | 1.13-2.59 |  | 0.61 | 0.56-0.67 |  | 0.23 |  | |  |  |  |  |  |  |
| any danger |  | 3.10 | 1.71-5.61 |  | 0.65 | 0.57-0.74 |  | 0.23 |  | 1.90 | 1.23-2.93 |  | 0.61 | 0.56-0.67 |  | 0.27 |  | |  |  |  |  |  |  |
| evacuated |  | 0.98 | 0.53-1.78 |  | 0.62 | 0.53-0.71 |  | 0.64 |  | 1.10 | 0.73-1.66 |  | 0.60 | 0.55-0.66 |  | 0.05 |  | |  |  |  |  |  |  |
|  |  |  |  |  |  |  |  |  |  |  |  |  |  |  |  |  |  | |  |  |  |  |  |  |
| Mississippi |  |  |  |  |  |  |  |  |  |  |  |  |  |  |  |  |  | |  |  |  |  |  |  |
| any illness |  | 3.49 | 1.41-8.60 |  | 0.60 | 0.52-0.69 |  | 0.72 |  | 3.35 | 1.64-6.84 |  | 0.61 | 0.55-0.66 |  | 0.15 |  | |  |  |  |  |  |  |
| any damage |  | 1.42 | 0.64-3.14 |  | 0.61 | 0.52-0.69 |  | 0.44 |  | 1.36 | 0.78-2.40 |  | 0.59 | 0.54-0.6.5 |  | 0.06 |  | |  |  |  |  |  |  |
| any danger |  | 1.23 | 0.56-2.72 |  | 0.61 | 0.52-0.69 |  | 0.73 |  | 2.37 | 1.45-3.89 |  | 0.63 | 0.57-0.69 |  | 0.65 |  | |  |  |  |  |  |  |
| evacuated |  | 0.72 | 0.30-1.75 |  | 0.62 | 0.53-0.71 |  | 0.71 |  | 1.13 | 0.66-1.92 |  | 0.60 | 0.54-0.65 |  | 0.07 |  | |  |  |  |  |  |  |
|  |  |  |  |  |  |  |  |  |  |  |  |  |  |  |  |  |  | |  |  |  |  |  |  |
| Isaac |  |  |  |  |  |  |  |  |  |  |  |  |  |  |  |  |  | |  |  |  |  |  |  |
| any illness |  | 3.33 | 1.61-6.90 |  | 0.65 | 0.55-0.75 |  | 0.35 |  | 4.36 | 2.56-7.41 |  | 0.68 | 0.62-0.74 |  | 0.34 |  | |  |  |  |  |  |  |
| any damage |  | 1.83 | 0.95-3.52 |  | 0.63 | 0.53-0.72 |  | 0.50 |  | 1.24 | 0.81-1.90 |  | 0.60 | 0.54-0.66 |  | 0.10 |  | |  |  |  |  |  |  |
| any danger |  | 2.76 | 1.46-5.22 |  | 0.67 | 0.58-0.76 |  | 0.19 |  | 1.91 | 1.25-2.92 |  | 0.64 | 0.58-0.69 |  | 0.73 |  | |  |  |  |  |  |  |
| evacuated |  | 0.74 | 0.37-1.47 |  | 0.61 | 0.52-0.71 |  | 0.57 |  | 1.26 | 0.81-1.96 |  | 0.59 | 0.53-0.66 |  | 0.16 |  | |  |  |  |  |  |  |
| *Adjusted for age, pregnancy status, race, and income | | | | | | | | | | | | | |  |  |  |  | |  |  |  |  |  |  |
